# Pink October and Breast Cancer in Brazil

**DOI:** 10.1055/s-0041-1739451

**Published:** 2021-11-16

**Authors:** Marcos Felipe Silva de Sá

**Affiliations:** 1 Editor-in-Chief RBGO Obstetrics and Gynecology

The National Cancer Institute of the Brazilian Ministry of Health has launched this October, as it has been done since 2010, the Pink October campaign. Since 1990, this campaign has been performed worldwide annually, when there is extensive dissemination through the media, social events and educational programs which aims to alert women and society about the importance of prevention and early diagnosis of breast cancer. There is also an important participation of medical societies and health professionals.

Excluding non-melanoma skin tumors, breast cancer is the tumor that most affects the Brazilian female population and represents ∼24.5% of all types of diagnosed neoplasms. In Brazil, in the 2020–2022 triennium, the estimated incidence is around 66,280 new cases of breast cancer per year, which represents a rate of 61.61 cases/100,000 women. In 2019, 18,068 deaths from breast cancer were recorded, being the leading cause of death from cancer among women. 1 2

In the United States, the incidence of breast cancer has been increasing by ∼3% per year, considering the 2012–2016 period. 3 In 2021, 281,550 new cases of invasive breast cancer and also 49,290 new cases of non- invasive cancer (in situ) are estimated. One out each eight American women will develop breast cancer during their lifetime. 4 In developing countries, the incidence has increased substantially in recent decades. 5 In 2020, more than 2.3 million women had the diagnosis of breast cancer around the world. 1

Considering the importance of this disease for public health, the United Nations have established as one of the Sustainable Development Goals, to reduce by a third the number of premature deaths from chronic noncommunicable diseases, including breast cancer, until year 2030. Projections by the National Cancer Institute of Brazil point to stability in mortality rates between 30 and 69 years until 2030, although these numbers are still far from the 30% reduction established by the United Nations. 1

Like many other types of cancer, it is known that prevention or early identification and rapid institution of tumor treatment for breast cancer lead to better therapeutic results and higher patient survival rates. In fact, campaigns to reduce breast cancer mortality rates can gain strength if services are expanded to provide quality prevention, early detection and timely treatment so that all women who need such services have quick access to them. In many countries, well-organized screening programs have led to a reduction in breast cancer mortality rates. 6 7 Considering the whole world, the incidence/mortality rate is 3.3. 8 In the United States, the breast cancer mortality rate has decreased and fallen 40% between 1989 to 2017, which represents ∼375,900 avoided deaths. 3

Unfortunatelly, in Brazil, despite these periodic campaigns, there has been an increase in incidence and mortality rates associated with breast cancer. 9 10 11 During these campaigns, a lot is focused on publicizing the need for screening for breast cancer, but the effective measures taken to facilitate the access of patients to medical care or imaging exams for this purpose are unclear. More than that, very little disclosure about the expected flow for patients, in a regionalized and hierarchical manner, for their referral to treatment and follow-up centers. There are not enough specialized services and mammography equipment to meet the demands. Diagnosis appointments take a long time and once diagnosed, a true “via crucis” begins for patients and health professionals until their effective referral to tertiary centers for treatment. As there are few service centers, patients are forced to long waits until they start the treatment.

In 2017, the main Brazilian specialty medical societies involved in breast cancer diagnosis and treatment programs recommended annual mammography as screening for breast cancer in women in the medium-risk population, i.e., those aged between 40 and 74 years. 12 They anticipated the ages for the screening examination based on a peculiarity of breast cancer in Brazil, different from other developed countries, a proportionally higher incidence in women between 40 and 50 years of age. 13 14 However, this suggestion was not implemented by the Ministry of Health, considering the lack of resources for this initiative. Mammography has been described as an effective method for the early detection of breast cancer, with a significant impact on patient survival, 15 although this impact varies in different populations with a clear decline in developing countries compared with developed countries. The differences in this impact have been attributed to late diagnosis and technological differences used in cancer therapy. 16 In Brazil, from 2000 to 2018, 40% of women between 50 and 69 years old, were diagnosed with cancer when the tumor was locally advanced or already metastatic. Furthermore, when analyzing patients' 10-year survival rates according to the tumor stage, the data showed how significant the worsening is when the tumor was treated in stages 3 and 4. 17 This suggests that the diagnosis when the tumor is already advanced is a consequence of the lack of guidance to patients for screening or delay in the referral of patients to treatment. 17

Data published by the Oncological Observatory in 2020 show that the average time to start the diagnostic procedure for breast cancer in Brazil was 38 days, ranging from 19 to 90 days between different states of the federation. In 29.7% of patients, the time elapsed between diagnosis and treatment ranged from 0–30 days; in 20.3% between 30–60 days, and in 24% the time was longer than 60 days. It is worth considering that despite these worrying numbers, they have been stable over the last decade. 18 We still do not have data for this period of the COVID-19 pandemic, but these statistics have certainly become much more worrisome. Breast cancer has increased its incidence among black and mixed-race individuals, especially in the poorest regions of Brazil 19 and this is exactly the population that most needs assistance in public health services. It is certainly this poorer population that makes the Brazilian statistics so negative with regard to breast cancer, since, on many occasions, they are left unattended in the search for this care.

Other factors must be considered for the improvement of breast cancer indicators. In Brazil, in 2020, according to National Cancer Institute data, around eight thousand cases of breast cancer were directly related to behavioral factors, such as alcohol consumption, overweight, not having breastfed and physical inactivity 1 . Therefore, in addition to care services to patients, the public health policies must pay attention to the need for permanent campaigns to publicize preventive measures for the population at risk for breast cancer.

The Pink October campaign will be senseless if there is no effective organization of the system to guide patients to seek a service adequately prepared for screening the disease and, above all, specialized services for the more agile treatment and monitoring of patients with a diagnosis or suspected diagnosis. The Brazilian Public Health Service (Brazilian SUS) has been implemented based on provision of organized services in a regionalized and hierarchical manner, but the lack of resources and inadequate management have shown important flaws in the service. The results achieved in the diagnosis and treatment of cancer of breast can be seen as a reflection of the system disorganization. Pink October needs to stop being just another fleeting and repetitive month of campaigning and effectively become a call for health managers to get truly involved to provide the results that society expects from the country's health authorities.
